# Chronic Water-Pipe Smoke Exposure Induces Injurious Effects to Reproductive System in Male Mice

**DOI:** 10.3389/fphys.2017.00158

**Published:** 2017-04-04

**Authors:** Badreldin H. Ali, Khalid A. Al Balushi, Mohammed Ashique, Asem Shalaby, Mohammed A. Al Kindi, Sirin A. Adham, Turan Karaca, Sumaya Beegam, Priya Yuvaraju, Abderrahim Nemmar

**Affiliations:** ^1^Department of Pharmacology, College of Medicine and Health Sciences, Sultan Qaboos UniversityAl Khod, Oman; ^2^Department of Pathology, College of Medicine and Health Sciences, Sultan Qaboos UniversityAl Khod, Oman; ^3^Department of Biology, College of Science, Sultan Qaboos UniversityAl Khod, Oman; ^4^Department of Histology-Embryology, Faculty of Medicine, University of TrakyaEdirne, Turkey; ^5^Department of Physiology, College of Medicine and Health Sciences, United Arab Emirates UniversityAl Ain, United Arab Emirates

**Keywords:** water-pipe smoke, mice, tobacco, testes, reproductive hormones

## Abstract

There is a global increase in the popularity of water-pipe tobacco smoking including in Europe and North America. Nevertheless, little is known about the male reproductive effects of water-pipe smoke (WPS), especially after long-term exposure. Here, we assessed effects of WPS exposure (30 min/day) in male mice for 6 months. Control mice were exposed to air-only for the same period of time. Twenty-four hours after the last exposure, testicular histopathology, and markers of inflammation and oxidative stress, and the tyrosine–protein kinase vascular endothelial growth factor receptor 1 (VEGFR1) were assessed in testicular homogenates. Moreover, plasma testosterone, estradiol, and luteinizing hormone (LH) concentrations were also measured. Chronic WPS exposure induced a significant decrease of testosterone and estradiol, and a slight but significant increase of LH. Glutathione reductase, catalase, and ascorbic acid were significantly decreased following WPS exposure. Plasma concentration of leptin was significantly decreased by WPS exposure, whereas that of tumor necrosis factor α and interleukin 6 was significantly increased. Histopathological analysis of the testes revealed the presence of a marked reduction in the diameter of the seminiferous tubules with reduced spermatogenesis. Transmission electron microscopy examination showed irregular thickening and wrinkling of the basement membranes with abnormal shapes and structures of the spermatozoa. VEGFR1 was overexpressed in the testis of the mice exposed to WPS and was not detected in the control. The urine concentration of cotinine, the predominant metabolite of nicotine, was significantly increased in the WPS-exposed group compared with the control group. We conclude that chronic exposure to WPS induces damaging effects to the reproductive system in male mice. If this can be confirmed in humans, it would be an additional concern to an already serious public health problem, especially with the increased use of WPS use all over the world, especially in young adults.

## Introduction

Water-pipe smoking (WPS; also called *hookah, shisha*, Hubble bubble, and *narghila*) is a form of smoking which uses tobacco sweetened with either fruit or molasses sugar, which makes the smoke more aromatic than cigarette smoke. The tobacco in WPS is exposed to high heat from burning charcoal, and the smoke produced is equal or even more toxic than cigarette smoke (Centers for Disease Control and Prevention, 2016).

A typical WPS session lasts 30 min or up to several hours, and can expose the smoker to 100–200 times the volume of smoke of a single cigarette (Chaouachi, 2009, 2011). WPS is customarily used by people from either Middle Eastern or Asian community groups but is becoming increasingly popular in Western countries. A recent national adult tobacco survey among USA adults showed that the national prevalence of WPS use was 9.8 and 1.5% for current use (Salloum et al., 2015).

Even though the respiratory and extra-respiratory pathophysiological effects of cigarette smoking (CS) are well-studied, much less has been reported on WPS, and there is a general and erroneous perception among users that WPS is relatively free from the adverse effects of CS (Knishkowy and Amitai, 2005; Maziak, 2015).

We have recently shown that acute and subacute exposure to WPS causes lung inflammation and oxidative stress (Nemmar et al., 2013a, 2015a). Chronic WPS exposure, however, causes lung inflammation, and oxidative stress, as well as DNA damage, enlargement of alveolar spaces and ducts associated with impairment of lung function (Nemmar et al., 2016). On the cardiovascular system, we have demonstrated that 5 days and 1 month exposure to WPS causes heart inflammation and oxidative stress, and thrombotic events (Nemmar et al., 2013b, 2015b). More recently, we showed that chronic exposure to WPS increased blood pressure, relative heart weight, troponin I and brain natriuretic peptide in plasma, and induced hypoxemia, hypercapnia and thrombosis (Nemmar et al., 2017). Moreover, WPS caused cardiac oxidative stress, DNA damage and fibrosis (Nemmar et al., 2017).

Results on the effects of CS on male fertility are at variance (Soares and Melo, 2008). The majority of the published data indicate that it has several adverse effects on reproductive health in smokers and laboratory animals exposed to the cigarette smoke (Ahmadnia et al., 2007; Kovac et al., 2015). However, we are not aware, of any published reports on the possible actions of WPS on male reproduction in smokers or laboratory animals except for our recent report in which mice were exposed to WPS for 1 month (Ali et al., 2015). Nevertheless, no experimental data are available on the chronic (6 months) impact of exposure on the male reproductive system. Such experimental studies are important because they will provide biological plausibility to the possible long-term deleterious effects of WPS on the male reproductive system in humans. The study of the effects of WPS has proven to be difficult because most of the smokers are also either current or former cigarette smokers (Knishkowy and Amitai, 2005). Therefore, the aim of the present study was to assess the chronic (6 months) impact of WPS on the male reproductive system by analysing a comprehensive set of parameters including testicular immunohistochemistry and histopathology (by light and electron microscopy) and, inflammation and oxidative stress, and vascular endothelial growth factor receptor 1 (VEGFR1), a tyrosine–protein kinase known to be increased in cases of impaired spermatogenesis. Moreover, plasma testosterone, estrogen, and luteinizing hormone (LH) concentrations were also measured.

## Materials and methods

### Animals

BALB/ c male mice (~20 g, 8-weeks old), obtained from Taconic Farms Inc. Germantown, NY, USA, were maintained at the College of Medicine and Health Sciences animal house (United Arab Emirates University, UAEU) on a 12-h light–dark cycle (lights on at 6:00). The animals (*n* = 24) were housed in cages and given unrestricted pelleted food and tap water. This study was carried out in accordance with the recommendations of our Institutional Animal Care and Research Advisory Committee of the College of Medicine and Health Sciences, UAEU, as described previously in our publications on WPS (Nemmar et al., 2013b, 2016). The protocol was approved by our Institutional Animal Care and Research Advisory Committee of the College of Medicine and Health Sciences, UAEU. Following acclimation for 7 days, the mice were randomly divided into two equal groups: control and WPS-exposed. The mice were put in soft restraints and connected to the exposure tower (Nemmar et al., 2012, 2015a; Ali et al., 2015), and exposed to either WPS or air through their noses using a nose-only exposure system (InExpose System, Scireq, Canada). Mice were exposed to mainstream WPS exactly as described before (Nemmar et al., 2013b, 2016). The duration of the session was 30 min/day for 6 consecutive months. The control mice were treated in a similar manner and were exposed to filtered air for the same duration. By the end of the experiment one mouse died in the control group, and two in the WPS-exposed group (leaving 11 in the control group, and 10 in the experimental group). The experimental design involved replicating the experiment once (each with about half the number of animals in each group), separated by a week.

### Blood collection and tissue homogenization

Twenty–four hours after the end of the experimental period, the animals were weighed and then anesthetized with pentobarbital sodium (45 mg/kg) intraperitoneally. Blood (about 1.5 ml) was then obtained from the inferior vena cava in EDTA (4%) and spun at 900 *g* for 15 min at 4°C. Blood collection was carried out at the same time of day (8–9 AM) to avoid circadian variation in the reproductive hormones (Lucas and Eleftheriou, 1980). The plasma collected was kept frozen at −80°C pending biochemical analysis. Then the lung and testes from all mice were collected and rinsed with ice-cold PBS (pH 7.4). The testes were weighed and the right testis and the upper half of the left testis were immediately frozen at −80°C until biochemical and molecular studies. Half the left testis was homogenized in 0.1 M phosphate buffer pH 7.4 containing 0.15 M KCl, 0.1 mM EDTA, 1 mM DTT, and 0.1 mM phenylmethylsulfonyl fluoride at 4°C. The homogenates were spun at 4°C for 10 min at 3,000 *g* to remove cellular debris and the supernatants were kept for further analysis. The right testis was stored at −80°C and later used in certain tests (see below).

### Blood plasma analysis

The plasma concentrations of testosterone, luteinizing hormone (LH) and estradiol were measured by ELISA, and total cholesterol by a spectrophotometric method (Sigma–Aldrich, St. Louis, MO, USA), as described before (Ali et al., 2012). Tumor necrosis factor alpha (TNFα), interleukin 6 (IL6) and leptin concentrations were measured by ELISA methods using kits from R & D systems (Minneapolis, MN, USA).

### Urine analysis

The concentrations of cotinine in urine (ELISA kit from Creative Diagnostics, Shirley, NY, USA) were measured after the last exposure to either WPS or air on urine samples collected overnight using metabolic cages for urine collection.

### Testicular homogenate analysis

The concentration of testicular protein content was estimated by Bradford's method, as described before (Korpelainen et al., 1998). Oxidative stress was assessed by measuring the activities of superoxide dismutase (SOD), catalase, and glutathione reductase, as well as total ascorbic acid concentration using ELISA kits (BioVision, Milpitas, CA, USA). The activity of alkaline phosphatase was measured spectrophotometrically using a commercial kit (Human, Wiesbaden, Germany).

### Histology

A part of the right testis was taken from randomly selected five controls and six treated animals and placed first in Bouin's fluid for an hour (Tu et al., 2011; Dutta et al., 2012), and then transferred to 10% formalin, and processed as described before (Korpelainen et al., 1998). The H & E sections (6 μM) were examined by a specialist unaware of the treatments using a Olympus microscope (BX51, Tokyo, Japan). The mean seminiferous tubule diameter (MSTD) was calculated in μm. Spermatogenesis and testicular damage were evaluated histopathologically using Johnsen's mean testicular biopsy score (MTBS) criteria (Johnsen, 1970). A score of 1–10 was given to each tubule according to epithelial maturation (1, no cells; 2, Sertoli cells without germ cells; 3, only spermatogonia; 4, only a few spermatocytes; 5, many spermatocytes; 6, only a few early spermatids; 7, many early spermatids without differentiation; 8, few late spermatids; 9, many late spermatids; 10, full spermatogenesis). For these evaluations, MSTD and MTBS were calculated in 50 tubules of each testis using an Olympus CX31 microscope. At the same time, the testicular sections were observed and the mean numbers of spermatogonia, spermatocytes, spermatids, and Sertoli cells in each tubule were calculated (Karaca et al., 2015).

### Transmission electron microscopy

A part of the right testis (from the same five controls and six treated animals randomly selected above) was cut into pieces (2 × 2 mm) and fixed in 2.5% glutaraldehyde (pH = 7. 4) for 6–8 h at 4°C. They were washed and post fixed in 2% OsO_4_ for 1 h, at 4°C. The tissue was dehydrated through ascending grades of ethanol and embedded in araldite CY212. Semi thin sections (1 μm) were cut and stained with toluidine blue. Ultra-thin sections (60–70 nm) were cut and stained with uranyl acetate and alkaline lead citrate.

### Immunohistochemistry

Paraffin imbedded tissues were cut into three μm sections and stained immunohistochemically using (EnVision™ + Dual Link System-HRP (DAP+) a kit purchased from Dako (USA). The sections were deparaffinized by xylene, rehydrated in graded ethyl alcohol (100, 95, and 75%) and washed with tap water. Antigen retrieval was conducted by 1 mM Ethylenediaminetetraacetic acid (EDTA) (pH 9.0) in a 95°C water bath for 30–40 min. Tissue area on the slides was circled using a hydrophobic pin (Dako, USA). The activity of endogenous peroxidases was blocked by Dual Endogenous Enzyme Block (Dako, USA) for 10 min. The slides were washed in PBS twice and incubated with anti-VEGFR1 primary antibody (ab81321; Abcam, UK) in a dilution of 1:100 at 4°C overnight. The slides were washed twice in PBS prior to their incubation with Labeled Polymer-HRP (Dako, USA) as a secondary antibody for 30 min at room temperature. Another washing step in PBS was needed for 5 min, followed by incubation with substrate chromogen solution (20 μl DAB + chromogen diluted in 1 ml DAB + substrate buffer; Dako) for 5–10 min. The following step was counterstaining using hematoxylin solution, which was added for 1–2 min. Then dehydration of tissues was done using graded ethanol (75, 95, and 100%) and xylene. Finally, the slides were mounted using DPX (Di-n-butyl phthalate in Xylene; Sigma, USA). Tissues were visualized using a light microscope (Nikon H600L) with digital camera (DS-Ri2). The imaging software used was NIS Elements version 4.40. The negative control was prepared using exactly the same procedure but with the replacement of the primary antibody with PBS.

### Drugs, chemicals, and kits

Alkaline phosphatase kit was purchased from Human GmbH, Wiesbaden, Germany, and kits for catalase, glutathione reductase, and SOD from BioVision Incorporated, Milpitas, CA, USA. The kits for testosterone, LH and estradiol-17 beta were obtained from DRG Instruments, GmbH, Marburg, Germany. All other chemicals used were of the highest grade commercially available.

### Statistical analysis

Values reported are mean ± standard error of the mean (SEM). Statistical significance of the data was assessed by the unpaired Student's test, using GraphPad, Prism software, version 5. To determine whether parameters were normally distributed, the KD normality test was applied, and *p* < 0.05 were considered significant.

## Results

The 6 month exposure to WPS did not change significantly the body or testicular weight of mice (Table 1).

**Table 1:** **Some basic physiological indices of control mice and mice exposed to WPS for 30 min/day for 6 months**.

**Parameters**	**Air exposed**	**WPS treated**	***p*-value**
Initial Body Weight (g)	22.1 ± 1.0	24.2 ± 1.1	*p* > 0.05
Final Body Weight (g)	24.3 ± 0.9	24.7 ± 1.1	*p* > 0.05
Body Weight change (%)	12.6 ± 7.2	2.8 ± 3.9	*p* > 0.05
Total Testicular Weight (g)	0.18 ± 0.01	0.20 ± 0.007	*p* > 0.05
Relative Testicular Weight (%)	0.76 ± 0.03	0.81 ± 0.02	*p* > 0.05

*The values are mean ± SEM (from 10 to 11 mice in each group)*.

*Values in the table are means ± SEM (n = 10–11)*.

**Figure 1:**
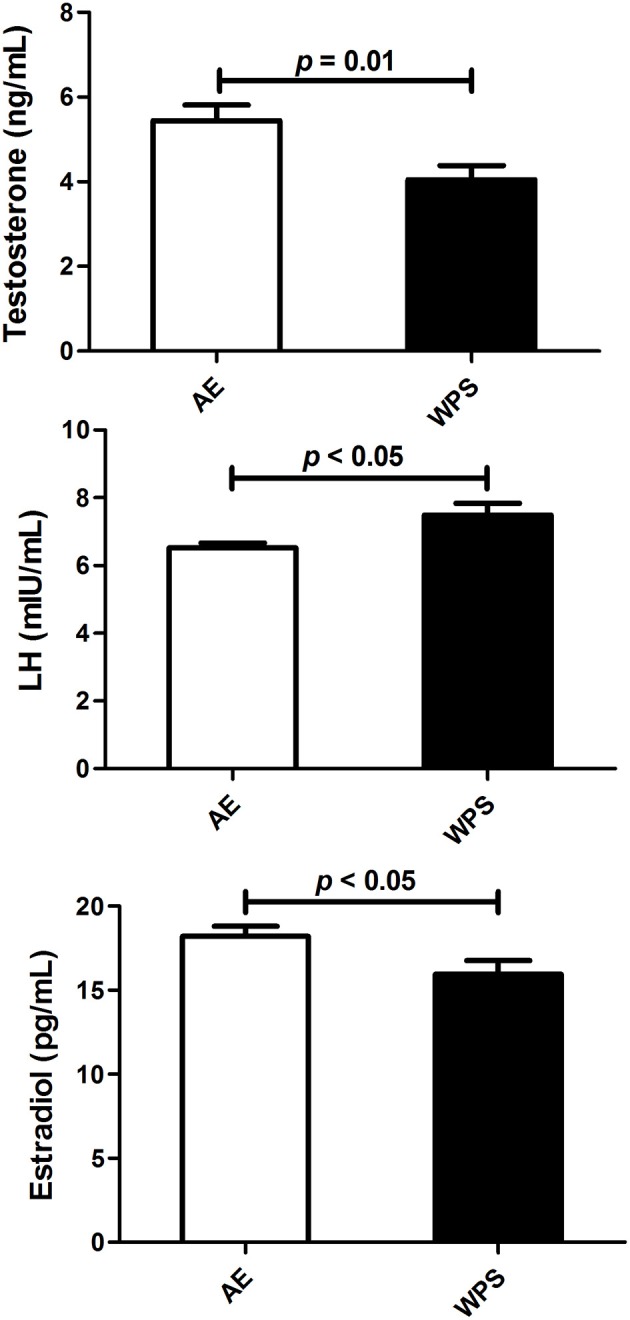
**The plasma concentrations of testosterone, luteinizing hormone (LH) and estradiol following air exposure (AE) of control mice (open columns), and water-pipe smoke (WPS) exposure for 30 min/day for 6 months (filled columns)**. Each column and vertical bar represent the mean ± SEM (*n* = 10–11 mice in each group).

As shown in Figure 1, the plasma concentrations of testosterone (*p* < 0.01) and estradiol (*p* < 0.05) were significantly lowered in mice exposed to WPS, compared with those that were exposed to normal air. The plasma concentration of LH was slightly but significantly elevated (*p* < 0.05).

**Figure 2:**
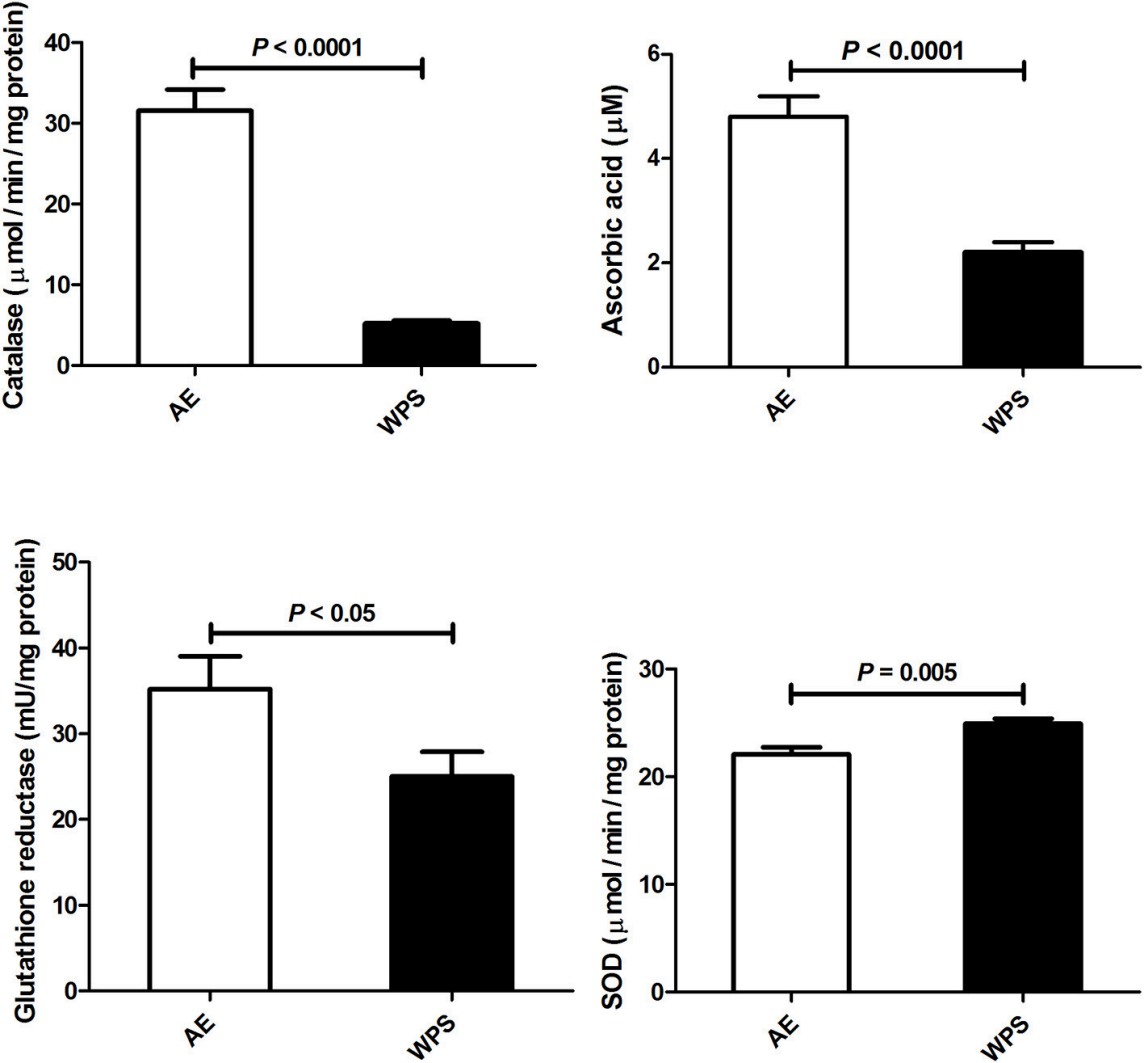
**Ascorbic acid concentration and catalase, glutathione reductase and superoxide dismutase (SOD) in testicular homogenates following air exposure (AE) of control mice (open columns), and water-pipe smoke (WPS) exposure for 30 min/day for 6 months (filled columns)**. Each column and vertical bar represent the mean ± SEM (*n* = 10–11 mice in each group). *P* < 0.05 was considered significant.

The 6 month exposure to WPS significantly lowered in the testicular concentrations and activities of indices of oxidative stress, those include glutathione reductase (*p* < 0.05), catalase (*p* < 0.0001), and total ascorbic acid (*p* < 0.0001). However, SOD activity was slightly but significantly increased by the WPS (Figure 2).

**Figure 3:**
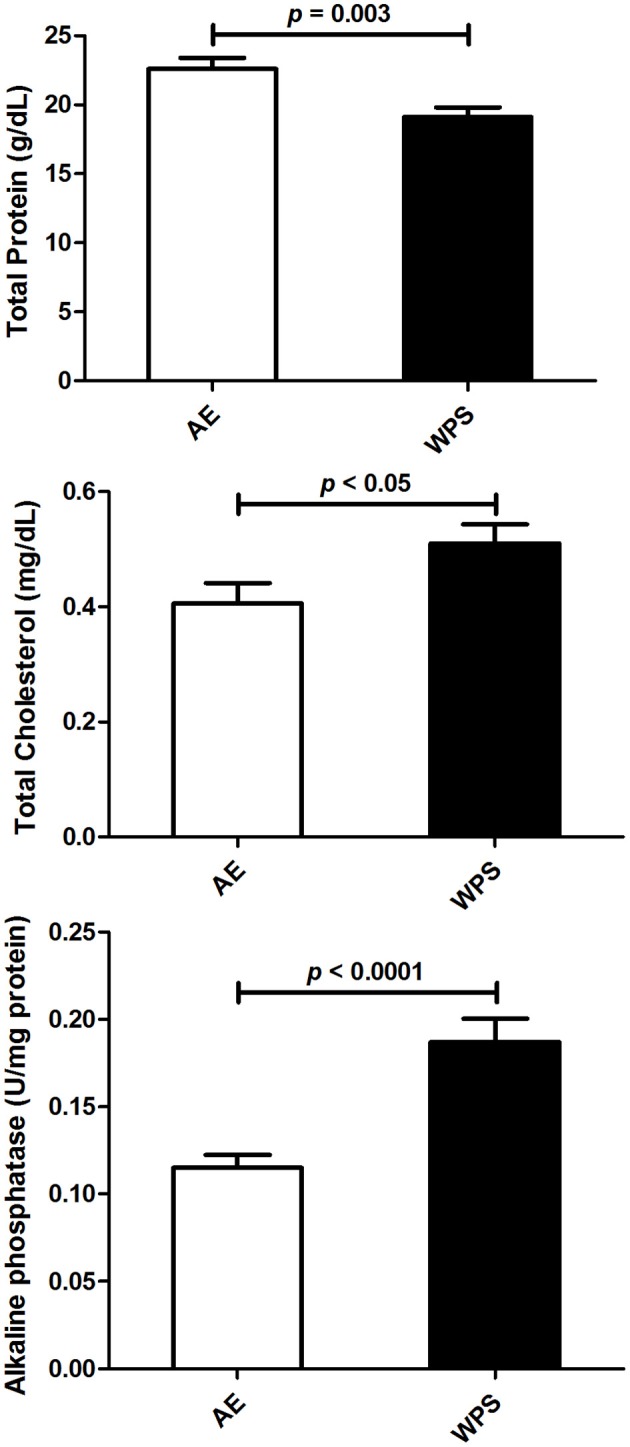
**The total protein and cholesterol concentrations, and the activity of alkaline phosphatase in testicular homogenates following air exposure (AE) of control mice (open columns), and water-pipe smoke (WPS) exposure for 30 min/day for 6 months (filled columns)**. Each column and vertical bar represent the mean ± SEM (*n* = 10–11 mice in each group). *P* < 0.05 was considered significant.

WPS exposure significantly decreased the testicular concentration of protein (*p* = 0.003), increased alkaline phosphatase activity (*p* < 0.001), and slightly but significantly elevated total cholesterol concertation (Figure 3).

**Figure 4:**
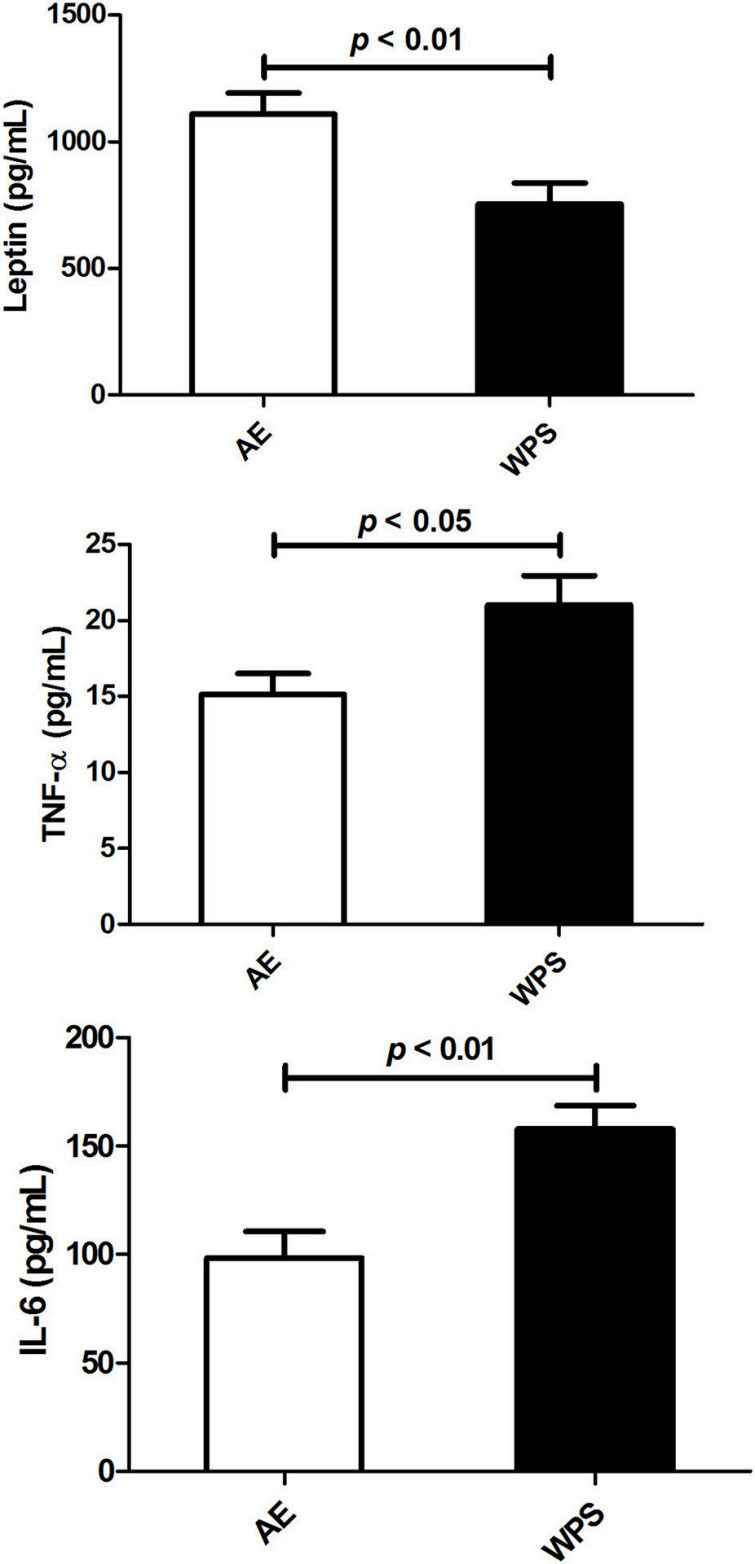
**The concentrations of leptin, tumor necrosis factor alpha (TNF-α) and interleukin 6 (IL-6) in plasma following air exposure (AE) of control mice (open columns), and water-pipe smoke (WPS) exposure for 30 min/day for 6 months (filled columns)**. Each vertical column and bar represent the mean ± SEM (*n* = 10–11 mice in each group). *P* < 0.05 was considered significant.

As shown in Figure 4, the plasma concentration of leptin was significantly decreased by WPS (*p* < 0.01), whereas that of TNF α was significantly increased (*p* < 0.05). The testicular concentration of IL-6 was also significantly increased (*p* < 0.01).

**Figure 5:**
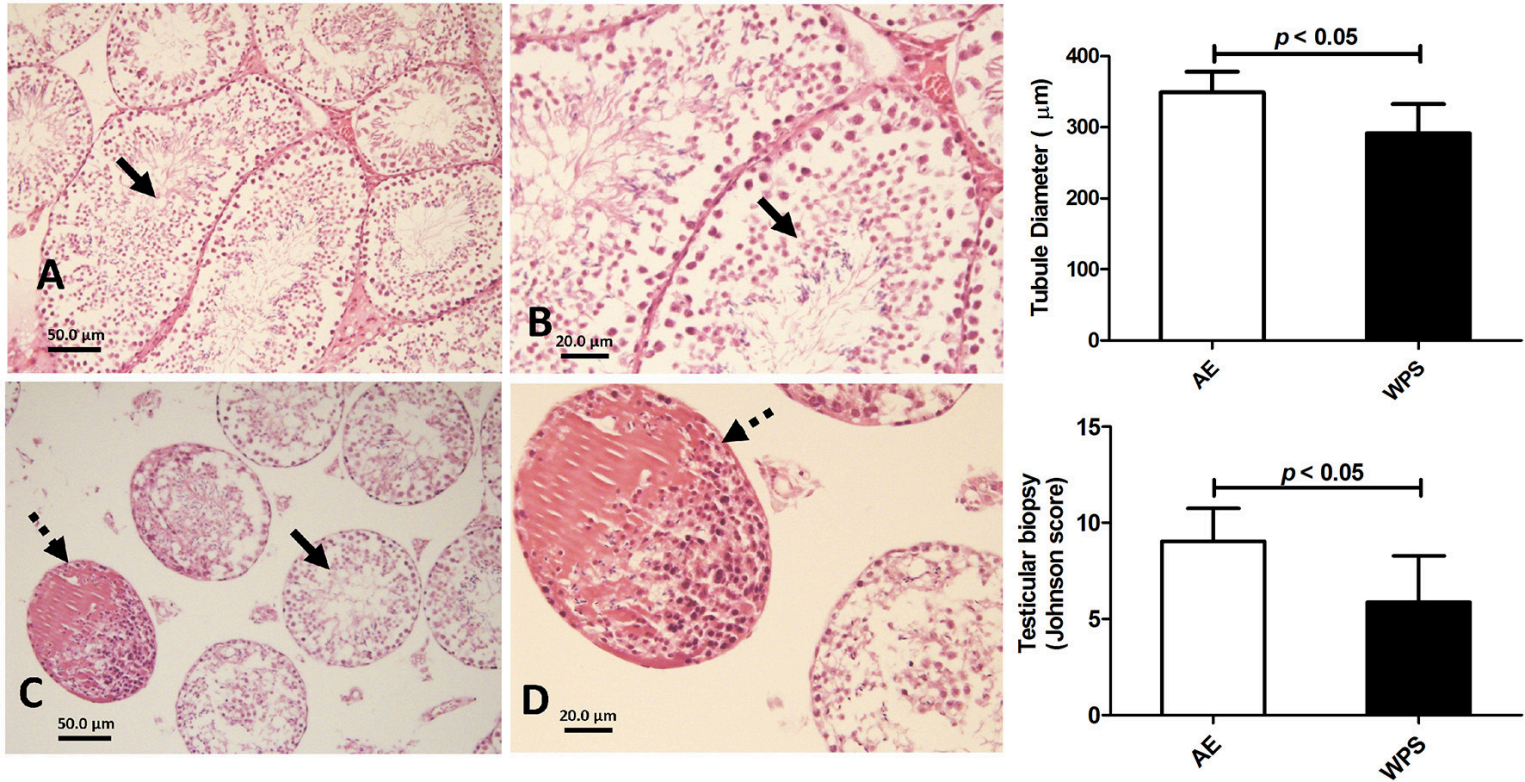
**Representative photomicrographs of a Hematoxylin-Eosin stained sections of testicular tissue obtained following air exposure (AE) of control mice (A,B)**, and water-pipe smoke (WPS) exposure **(C and D)** for 30 min/day for 6 months. Control mice showed normal spermatogenesis **(A,B)**, whereas those from mice exposed to WPS showed signs of damage **(A,B)**. The tubule diameter and the Johnson's score were both significantly decreased by the exposure to WPS (*p* < 0.05).

Figure 5 depicts the histology of the testicular tissues in mice exposed to either air (control) or WPS. The Figures 5A,B for control animals show photomicrographs of normal testicular structure with normal diameter and spermatogenesis (thick arrows, The Figures 5C,D for WPS-exposed mice show photomicrographs of testes with a marked reduction in the diameter of the seminiferous tubules with reduced spermatogenesis (thick arrows). Some tubules show destroyed lining and deposition of eosinophilic material in the lumen of seminiferous tubules (dashed arrows). The Johnsen score and the diameter of the tubules in the seminiferous tubules from the WPS-exposed mice were significantly decreased when compared with the normal values obtained from mice exposed to air only.

**Figure 6:**
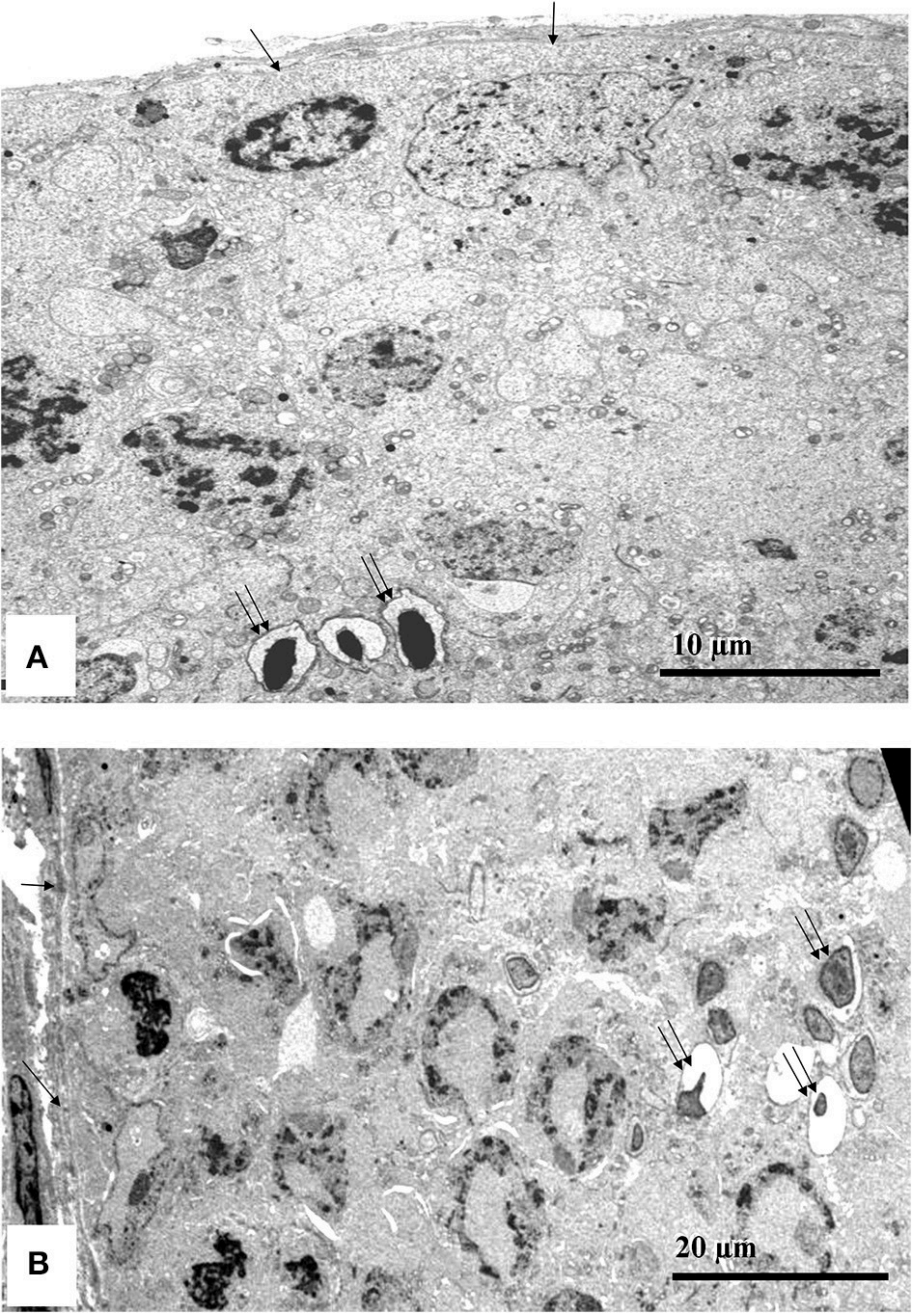
**Transmission electron microscopy testicular tissue photomicrographs from a representative control mouse (exposed to air only, A)** and a representative mouse exposed to water-pipe smoke (WPS, **B**) for 30 min/day for 6 consecutive months. The photomicrograph in the control group **(A)** showed normal basement membranes (single arrows) and normal structure of the spermatozoa (double arrows). The electron micrograph of the WPS group **(B)** showed irregular thickening and wrinkling of the basement membranes (single arrow) with abnormal shapes and structures of the spermatozoa (double arrows).

Figure 6 shows the transmission electron microscopy photomicrographs of the testicular tissues in mice exposed to either air (control) or WPS. The testes in the control group showed normal basement membranes (single arrows) and normal structure of the spermatozoa (double arrows). The electron micrograph of the WPS group showed irregular thickening and wrinkling of the basement membranes (single arrow) with abnormal shapes and structures of the spermatozoa (double arrows).

**Figure 7:**
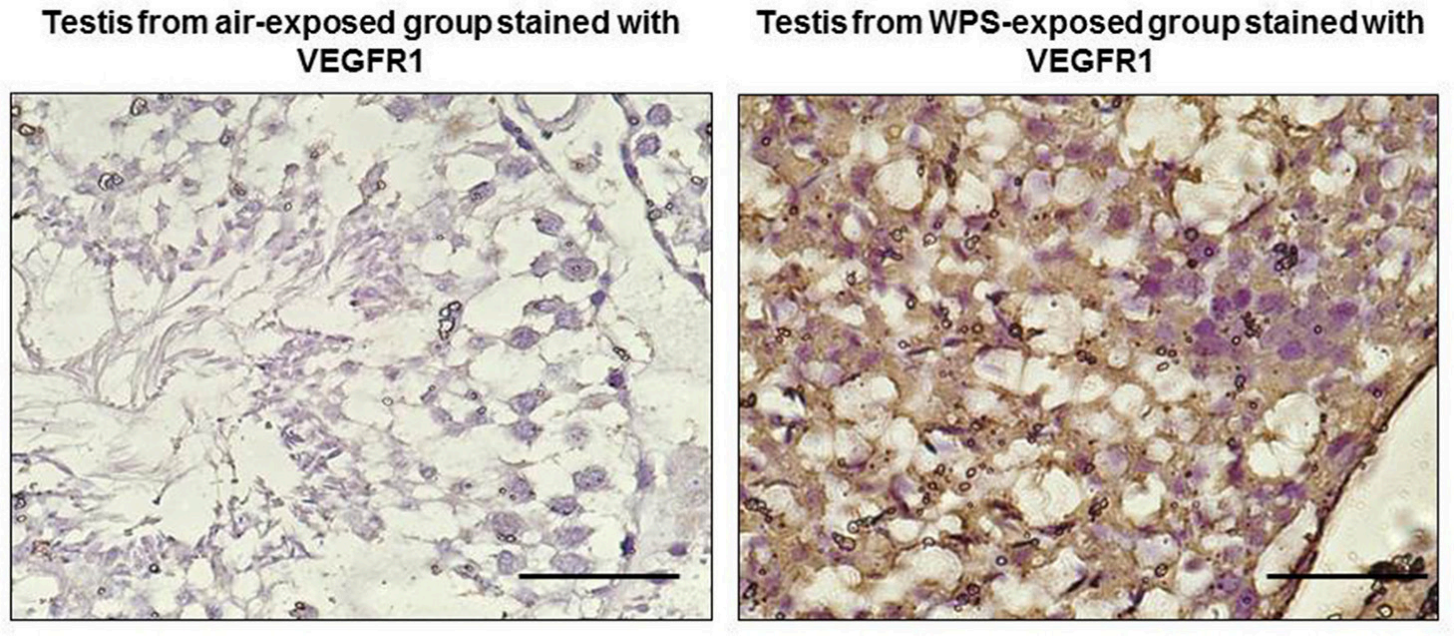
**Representative images for the expression of the vascular endothelial growth factor receptor 1 (VEGFR1) in testicular tissues in control mice exposed to air, and mice exposed daily for 30 min to water-pipe smoke (WPS) for 6 months**. VEGFR1 was overexpressed in the testes of the mice treated with WPS, and was not detected in the control not treated mice. The scale bar equals 50 μm.

The results obtained from the immunohistochemistry staining of the testis revealed that VEGFR1 was overexpressed in the testis of the mice exposed to WPS, but was not detected in control mice exposed to air only (Figure 7).

**Figure 8:**
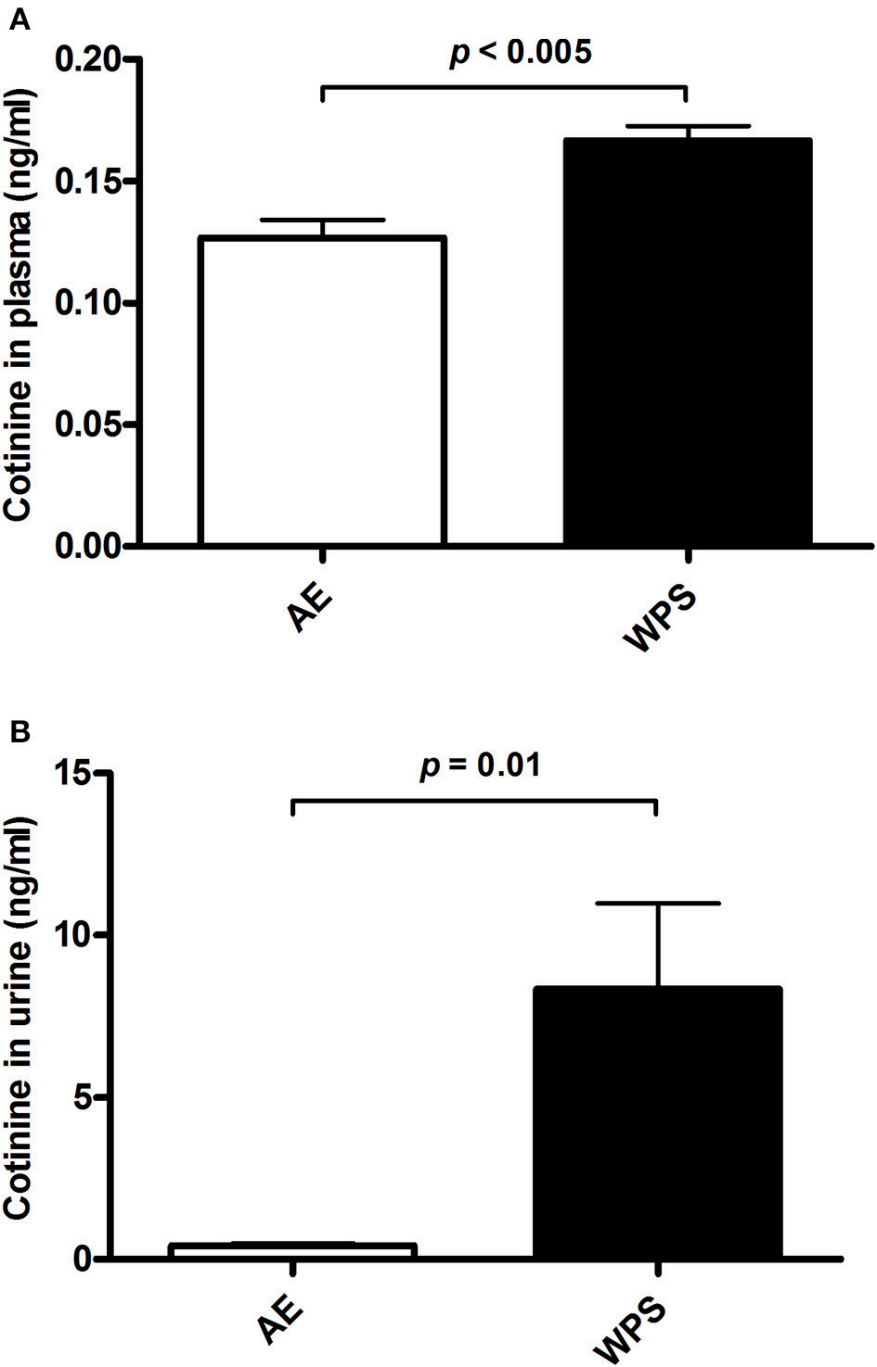
**The concentrations of cotinine in plasma (A)** and urine **(B)** following air exposure (AE) of control mice (open columns), and mice exposed to water-pipe smoke (WPS) for 30 min/day for 6 months (filled columns). Each vertical column and bar represent the mean ± SEM (*n* = 6 mice in each group). *p* < 0.05 was considered significant.

The concentration of cotinine was significantly increased in the plasma and urine of mice exposed to WPS for 6 months compared with those exposed to air (Figure 8).

## Discussion

In this work, exposure of mice to WPS for 6 months significantly lowered most of the protective antioxidant indices in the testes, and reduced the plasma concentrations of testosterone, estradiol, and slightly but significantly increased that of LH. VEGFR1 was overexpressed in the testes of the mice exposed to WPS and was not detected in the controls. A previous study showed that VEGF and its receptors VEGFR1 and VEGFR2 were expressed in the Leydig cells of the testes beside their usual expression in endothelial cells, and VEGFR1 in particular was shown to be expressed in certain spermatogenic cells (15). A more recent study showed that testes ischemia caused an increase in VEGFR1 mRNA which led to histological damage and reduced spermatogenic activity (Minutoli et al., 2012). Our present finding indicates that the increase in VEGFR1 levels in testicular tissues from WPS - exposed mice was a result of testicular damage, and may suggests an indicator of infertility. Since VEGFR1 is the main tyrosine kinase related to reduced spermatogenic activity in the testicular cells (Minutoli et al., 2012), it could be inferred that the WPS exposure may have caused an increase in the *FLT1* gene expression as seen by the increase in VEGFR1 protein which is related to infertility (15)

However, these actions were not accompanied by any significant change in the body or testicular weight. This suggests that the exposure of male mice to WPS for 6 months causes more severe adverse actions on the reproductive system of mice than those reported following a 1-month exposure (Ali et al., 2015). If this can be confirmed in humans, it would be an additional serious public health concern, especially with the increased use of WPS all over the world, especially in young adults.

This work examined the actions of WPS on the oxidant system in testicular homogenates. Enzymatic and non-enzymatic testicular antioxidants can function to terminate the regeneration of reactive oxygen species (ROS) in the oxidation of fats, and both inhibition and activation of proteins. Testicular oxidative stress is thought to be behind several cases of reproductive toxicities and infertility in humans and animals (Aitken and Roman, 2008; Turner and Lysiak, 2008). We found that the activities of the testicular antioxidant enzymes (e.g., glutathione reductase, CAT, but not SOD) and non-enzymatic antioxidant (e.g., total ascorbic acid) were significantly decreased in the WPS-exposed mice, when compared with the antioxidants in the air-exposed (control) group. It is established that those antioxidants are involved in the recycling of the detrimental effects of free radicals and free forms of oxygen (Noblanc et al., 2011; Nathan and Cunningham-Bussel, 2013). We have recently reported significant reductions in the testicular antioxidants in mice exposed to WPS for 1 month (Ali et al., 2015). A more marked reduction in these antioxidants has been observed in the current experiment with a longer exposure time. This action indicates that these affected testes would be susceptible to the adverse and potentially toxic actions of WPS and other xenobiotics, resulting in impaired cell function, cell death, and infertility. Administration of nicotine (0.5 and 1 mg/kg orally) to rats for 30 days has been shown to significantly lower many parameters of the antioxidant profile in plasma (Oyeyipo et al., 2013, 2014). The significant diminutions that we have observed in testicular antioxidants in mice exposed to WPS for 180 days could be due to nicotine solely, or/and to other components in the WPS, and this warrants further studies. It is well known that perinatal and postnatal nicotine by itself can interfere with normal sexual development in humans and rodents, probably through disruption of sexual hormones (Gyekis et al., 2010).

As far as we are aware, there are no reports in the literature on the effect of WPS on reproductive hormones except for our previous study in which we exposed mice to WPS for 30 days (Ali et al., 2015). In this work using 6 months of exposure, statistically significant decreases in plasma testosterone and estradiol concentrations were found, probably suggesting an inhibition in the hormone synthesis pathways and/or diminished responsiveness of Leydig cells to LH. Decreased testosterone level is expected to lead eventually to suppression of reproductive activities. Contrary to the previous work, however, LH concentration was slightly but significantly elevated (Ali et al., 2015). The latter effect has also been reported before in mice following arsenic-induced testicular toxicity (Souza et al., 2016). The increased concentration of LH with diminished circulatory testosterone levels in experimental mice may suggest an intact pituitary–testicular axis (Souza et al., 2016). The literature on the effect of cigarette smoking on testosterone is at variance, as shown in a recent review article (Zhao et al., 2016). Most of the published papers found that smokers had higher mean testosterone than non-smokers in men, and no clear effect on women. This has been explained by hypothesizing that nicotine and/or its metabolites have a common disposal pathway with androgenic hormones, and so can competitively inhibit androgen disposal (Zhao et al., 2016). However, in our work, the urinary excretion of nicotine metabolite was significantly enhanced, and we also found slight but significant decreases in both testosterone and estradiol concentrations in plasma, and no significant effect on LH. Our results are in agreement, however, with a recent report which found that rats exposed to cigarette smoke, had lower testosterone level than the controls (Aprioku and Ugwu, 2016). These findings are also difficult to reconcile with the small but significant increase in total cholesterol in the testes.

In several studies in cigarette smokers, increased circulatory levels of reproductive hormones have been reported, and it has been suggested that this action depends on several factors that include the number of cigarette packs smoked, and the duration of smoking (Jeng et al., 2014). As far as we are aware, no similar data have been obtained after using WPS in humans. However, the decrease in estradiol plasma concentration after WPS exposure was different from previous epidemiological reports in human smokers, which indicated enhanced endogenous estrogen concentration (Barrett-Connor and Khaw, 1987; Jeng et al., 2014). The reasons for the discrepancy are not known.

In this work, WPS exposure for 6 months significantly decreased the plasma concentration of the hormone leptin. Leptin is now established to play an important role as a neuroendocrine mediator in the reproductive system, by regulating different elements of the hypothalamic–pituitary–gonadal axis (Vázquez et al., 2015), and decreased leptin levels in plasma are known to be linked to lower fertility in both male and female subjects. In the present work, WPS exposure for 6 months resulted in a significant decrease in leptin plasma concentration, an action that is consistent with the decreased reproductive hormones in plasma, and the significant lowering of the antioxidant parameters in testicular homogenates. The reduction in the plasma leptin level after CS has been suggested to be linked to increased concentration of plasma catecholamines (Ali et al., 2012). Conversely, it has been reported that nicotine in CS actually raises plasma leptin concentration (Ali et al., 2012). In this work, there was no clear association between the observed reduced leptin concentration and body weight, as expected. The percentage increase in body weight of mice exposed to WPS was less than in the normal control, but this was not statistically significant due to the wide variations in the results, and the relatively small sample size used. Nicotine is established to increase the body's energy expenditure and could reduce appetite, which is why smokers have lower body weight than non-smokers.

We measured here the testicular concentration of cholesterol in mice exposed to WPS and to air because cholesterol is established to be the starting point of steroid hormone synthesis, and a modulator of male and female reproductive physiology. However, no significant effect on cholesterol testicular concentration was detected. A similar finding was reported before in male chickens treated with the nitrofuran antimicrobial agent furazolidone (Ali et al., 1984), probably suggesting that testicular total cholesterol concentration might not be as sensitive as other markers of testicular damage. The significant increase in testicular alkaline phosphate activity, as well as the slight but significant decrease in total protein following WPS exposure represents another evidence of testicular cells disruption.

Some of our results showed a relatively high degree of variability between the air–exposed (controls) and the WPS–exposed groups. These variations may be ascribed to the variation among the relatively small number of mice used in each group (*n* = 10–11), environmental (seasonal) changes, or other unknown factors. Also, some values reported here (viz. for ascorbic acid and LH) are different from that in our previous paper where mice were exposed to WPS for 1 month (Ali et al., 2015). This may be due to the fact that ascorbic acid was measured by an HPLC method in our previous paper (Ali et al., 2015), and by an ELISA method in the present work. The variation in the control value of LH may be due to seasonal changes, or to other unknown factor.

No noticeable gross or histological changes in the testes of mice exposed to WPS for 1 month were observed (Ali et al., 2015). However, after 6 months of exposure to WPS, histopathological analysis of the testes revealed the presence of a marked reduction in the diameter of the seminiferous tubules with reduced spermatogenesis. Transmission electron microscopy examination showed irregular thickening and wrinkling of the basement membranes with abnormal shapes and structures of the spermatozoa. VEGFR1 was overexpressed in the testis of the mice exposed to WPS and was not detected in the control. This may suggest that the level of WPS insult on the testes was not enough to induce light microscopic alterations in exposure of mice to WPS for 1 month, but was sufficient to produce clear histopathological changes after exposure to WPS for 6 months.

Cotinine, the predominant metabolite of nicotine, has a long half-life (20 vs. 2 h for nicotine), and is, consequently, used as a biomarker for tobacco smoke exposure (Mattes et al., 2014). Here, we found a significant increase of cotinine in plasma and urine of mice exposed to WPS compared with those exposed to air. The overall concentrations of cotinine were much higher in urine than in plasma. Moreover, the percentage increase of cotinine in in the urine was much greater than that observed in plasma of the WPS group. The latter suggests that cotinine produced from the metabolism of nicotine is rapidly eliminated in the urine.

It can be concluded that, exposure of mice to WPS for 6 months induced several alterations, including testicular oxidative stress and significant decreases in plasma testosterone and estradiol concentrations, in addition to some light- and electron microscopic histopathological changes in the testes. Moreover, we found a significant increase of cotinine in the urine of mice exposed chronically to WPS. More studies on the functional consequences of WPS exposure on semen, and on the effect of extended duration of exposure to WPS, and the identification of the chemicals in WPS that may be responsible for these adverse effects are needed.

## Ethics statement

This project was reviewed and approved by our Institutional Review Board of the United Arab Emirates University, College of Medicine and Health Sciences, and experiments were performed in accordance with protocols approved by the Institutional Animal Care and Research Advisory Committee.

## Author contributions

All authors have read and approved the manuscript. BA and AN designed, planned, supervised the experiments and wrote the article. KA, MA, AS, MAA, SA, TK, SB, and PY performed the experiments.

## Funding

This work was supported by Sultan Qaboos University (IG/MED/DEAN/15/1, in Oman) and the United Arab Emirate University [CMHS, UPAR (31M189) and Center-based interdisciplinary (31R052) grants, in the United Arab Emirates].

### Conflict of interest statement

The authors declare that the research was conducted in the absence of any commercial or financial relationships that could be construed as a potential conflict of interest.

## References

[B1] Ahmadnia H. Ghanbari M. Moradi M. R. Khaje-Dalouee M. (2007). Effect of cigarette smoke on spermatogenesis in rats. Urol. J. 4, 159–163. 17987579

[B2] Aitken R. J. Roman S. D. (2008). Antioxidant systems and oxidative stress in the testes. Adv. Exp. Med. Biol. 636, 154–171. 10.1007/978-0-387-09597-4_9 19856167

[B3] Ali B. H. Adham S. A. Al Balushi K. A. Shalaby A. Waly M. I. Manoj P.. (2015). Reproductive toxicity to male mice of nose only exposure to water- pipe smoke. Cell Physiol. Biochem. 35, 29–37. 10.1159/000369672 25547785

[B4] Ali B. H. Al-Lawati I. Beegam S. Ziada A. Al S. S. Nemmar A.. (2012). Effect of *Hibiscus sabdariffa* and its anthocyanins on some reproductive aspects in rats. Nat. Prod. Commun. 7, 41–44. 22428240

[B5] Ali B. H. Homeida A. M. Knifton A. (1984). The effect of furazolidone on fertility of male chickens. Comp. Biochem. Physiol. C 78, 43–47. 10.1016/0742-8413(84)90045-8 6146481

[B6] Aprioku J. S. Ugwu T. C. (2016). Tobacco smoke exposure induces irreversible alteration of testicular function in prepubertal rats. J. Basic Clin. Physiol. Pharmacol. 27, 577–584. 10.1515/jbcpp-2015-0153 27387331

[B7] Barrett-Connor E. Khaw K. T. (1987). Cigarette smoking and increased endogenous estrogen levels in men. Am. J. Epidemiol. 126, 187–192. 10.1093/aje/126.2.187 3605047

[B8] Chaouachi K. (2009). Hookah (Shisha, Narghile) smoking and environmental tobacco smoke (ETS). A critical review of the relevant literature and the public health consequences. Int. J. Environ. Res. Public Health 6, 798–843. 10.3390/ijerph6020798 19440416 PMC2672364

[B9] Chaouachi K. (2011). More rigor needed in systematic reviews on “waterpipe” (hookah, narghile, shisha) smoking. Chest 139, 1250–1251. 10.1378/chest.10-2864 21540226

[B10] Centers for Disease Control Prevention (2016). Smoking and Tobacco Use-Data and Statistics-Hookahs National Center for Chronic Disease Prevention and Health Promotion Office on Smoking and Health. Paris Available online at: https://www.cdc.gov/tobacco/data_statistics/fact_sheets/tobacco_industry/hookahs/

[B11] Dutta D. Park I. Mills N. C. (2012). Fixation temperature affects DNA integrity in the testis as measured by the TUNEL assay. Toxicol. Pathol. 40, 667–674. 10.1177/0192623311436182 22298796

[B12] Gyekis J. Anthony K. Foreman J. E. Klein L. C. Vandenbergh D. J. (2010). Perinatal nicotine exposure delays genital development in mice. Reprod. Toxicol. 29, 378–380. 10.1016/j.reprotox.2010.01.002 20080174

[B13] Jeng H. A. Chen Y. L. Kantaria K. N. (2014). Association of cigarette smoking with reproductive hormone levels and semen quality in healthy adult men in Taiwan. J. Environ. Sci. Health A Tox Hazard. Subst. Environ. Eng. 49, 262–268. 10.1080/10934529.2014.846195 24279617

[B14] Johnsen S. G. (1970). Testicular biopsy score count–a method for registration of spermatogenesis in human testes: normal values and results in 335 hypogonadal males. Hormones 1, 2–25. 10.1159/000178170 5527187

[B15] Karaca T. Demirtas S. Karaboga I. Ayvazz S. (2015). Protective effects of royal jelly against testicular damage in streptozotocin-induced diabetic rats. Turk. J. Med. Sci. 45, 27–32. 10.3906/sag-1311-103 25790526

[B16] Knishkowy B. Amitai Y. (2005). Water-pipe (narghile) smoking: an emerging health risk behavior. Pediatrics 116, e113–e119. 10.1542/peds.2004-2173 15995011

[B17] Korpelainen E. I. Karkkainen M. J. Tenhunen A. Lakso M. Rauvala H. Vierula M.. (1998). Overexpression of VEGF in testis and epididymis causes infertility in transgenic mice: evidence for nonendothelial targets for VEGF. J. Cell Biol. 143, 1705–1712. 10.1083/jcb.143.6.1705 9852161 PMC2132976

[B18] Kovac J. R. Khanna A. Lipshultz L. I. (2015). The effects of cigarette smoking on male fertility. Postgrad. Med. 127, 338–341. 10.1080/00325481.2015.1015928 25697426 PMC4639396

[B19] Lucas L. A. Eleftheriou B. E. (1980). Circadian variation in concentrations of testosterone in the plasma of male mice, a difference between BALB/cBy and C57BL/6By inbred strains. J. Endocrinol. 87, 37–46. 10.1677/joe.0.0870037 7430915

[B20] Mattes W. Yang X. Orr M. S. Richter P. Mendrick D. L. (2014). Biomarkers of tobacco smoke exposure. Adv. Clin. Chem. 67, 1–45. 10.1016/bs.acc.2014.09.001 25735858

[B21] Maziak W. (2015). Rise of waterpipe smoking. BMJ 350:h1991. 10.1136/bmj.h1991 25888390

[B22] Minutoli L. Antonuccio P. Squadrito F. Bitto A. Nicotina P. A. Fazzari C.. (2012). Effects of polydeoxyribonucleotide on the histological damage and the altered spermatogenesis induced by testicular ischaemia and reperfusion in rats. Int. J. Androl. 35, 133–144. 10.1111/j.1365-2605.2011.01194.x 21651579

[B23] Nathan C. Cunningham-Bussel A. (2013). Beyond oxidative stress: an immunologist's guide to reactive oxygen species. Nat. Rev. Immunol. 13, 349–361. 10.1038/nri3423 23618831 PMC4250048

[B24] Nemmar A. Al-Salam S. Yuvaraju P. Beegam S. Yasin J. Ali B. H. (2017). Chronic exposure to water-pipe smoke induces cardiovascular dysfunction in mice. Am. J. Physiol. Heart Circ. Physiol. 312, H329–H339. 10.1152/ajpheart.00450.2016 27940964

[B25] Nemmar A. Al Hemeiri A. Al Hammadi N. Yuvaraju P. Beegam S. Yasin J.. (2015a). Early pulmonary events of nose-only water pipe (shisha) smoking exposure in mice. Physiol. Rep. 3:e12258. 10.14814/phy2.12258 25780090 PMC4393146

[B26] Nemmar A. Al-Salam S. Yuvaraju P. Beegam S. Yasin J. Ali B. H. (2016). Chronic exposure to water-pipe smoke induces alveolar enlargement, DNA damage and impairment of lung function. Cell Physiol. Biochem. 38, 982–992. 10.1159/000443050 26938718

[B27] Nemmar A. Raza H. Subramaniyan D. John A. Elwasila M. Ali B. H.. (2012). Evaluation of the pulmonary effects of short-term nose-only cigarette smoke exposure in mice. Exp. Biol. Med. 237, 1449–1456. 10.1258/ebm.2012.012103 23354403

[B28] Nemmar A. Raza H. Yuvaraju P. Beegam S. John A. Yasin J.. (2013a). Nose-only water-pipe smoking effects on airway resistance, inflammation and oxidative stress in mice. J. Appl. Physiol. 115, 1316–1323. 10.1152/japplphysiol.00194.2013 23869065

[B29] Nemmar A. Yuvaraju P. Beegam S. Ali B. H. (2015b). Short-term nose-only water-pipe (shisha) smoking exposure accelerates coagulation and causes cardiac inflammation and oxidative stress in mice. Cell Physiol. Biochem. 35, 829–840. 10.1159/000369741 25634761

[B30] Nemmar A. Yuvaraju P. Beegam S. John A. Raza H. Ali B. H. (2013b). Cardiovascular effects of nose-only water-pipe smoking exposure in mice. Am. J. Physiol. Heart Circ. Physiol. 305, H740–H746. 10.1152/ajpheart.00200.2013 23812392

[B31] Noblanc A. Kocer A. Chabory E. Vernet P. Saez F. Cadet R.. (2011). Glutathione peroxidases at work on epididymal spermatozoa: an example of the dual effect of reactive oxygen species on mammalian male fertilizing ability. J. Androl. 32, 641–650. 10.2164/jandrol.110.012823 21441427

[B32] Oyeyipo I. P. Raji Y. Bolarinwa A. F. (2013). Nicotine alters male reproductive hormones in male albino rats: the role of cessation. J. Hum. Reprod. Sci. 6, 40–44. 10.4103/0974-1208.112380 23869150 PMC3713576

[B33] Oyeyipo I. P. Raji Y. Bolarinwa A. F. (2014). Antioxidant profile changes in reproductive tissues of rats treated with nicotine. J. Hum. Reprod. Sci. 7, 41–46. 10.4103/0974-1208.130823 24829530 PMC4018797

[B34] Salloum R. G. Thrasher J. F. Kates F. R. Maziak W. (2015). Water pipe tobacco smoking in the United States: findings from the National Adult Tobacco Survey. Prev. Med. 71, 88–93. 10.1016/j.ypmed.2014.12.012 25535678 PMC4423406

[B35] Soares S. R. Melo M. A. (2008). Cigarette smoking and reproductive function. Curr. Opin. Obstet. Gynecol. 20, 281–291. 10.1097/GCO.0b013e3282fc9c1e 18460944

[B36] Souza A. C. Marchesi S. C. Ferraz R. P. Lima G. D. de Oliveira J. A. Machado-Neves M. (2016). Effects of sodium arsenate and arsenite on male reproductive functions in Wistar rats. J. Toxicol. Environ. Health A 79, 274–286. 10.1080/15287394.2016.1150926 27029432

[B37] Tu L. Yu L. Zhang H. (2011). Morphology of rat testis preserved in three different fixatives. J. Huazhong Univ. Sci. Technol. Med. Sci. 31, 178–180. 10.1007/s11596-011-0247-0 21505980

[B38] Turner T. T. Lysiak J. J. (2008). Oxidative stress: a common factor in testicular dysfunction. J. Androl. 29, 488–498. 10.2164/jandrol.108.005132 18567643

[B39] Vázquez M. J. Romero-Ruiz A. Tena-Sempere M. (2015). Roles of leptin in reproduction, pregnancy and polycystic ovary syndrome: consensus knowledge and recent developments. Metabolism 64, 79–91. 10.1016/j.metabol.2014.10.013 25467843

[B40] Zhao J. Leung J. Y. Lin S. L. Schooling C. M. (2016). Cigarette smoking and testosterone in men and women: a systematic review and meta-analysis of observational studies. Prev. Med. 85, 1–10. 10.1016/j.ypmed.2015.12.021 26763163

